# Ultracompact Multielectrode Array for Neurological Monitoring

**DOI:** 10.3390/s19102286

**Published:** 2019-05-17

**Authors:** Ming-Yuan Cheng, Ramona B. Damalerio, Weiguo Chen, Ramamoorthy Rajkumar, Gavin S. Dawe

**Affiliations:** 1 Institute of Microelectronics, Agency for Science, Technology and Research, Singapore 138634, Singapore; damaleriom@ime.a-star.edu.sg (R.B.D.); chenwg@ime.a-star.edu.sg (W.C.); 2 Department of Pharmacology, Yong Loo Lin School of Medicine, National University Health System, National University of Singapore, Singapore 117600, Singapore; rajkumar.sai@gmail.com (R.R.); gavin_dawe@nuhs.edu.sg (G.S.D.)

**Keywords:** biopackaging, lead transfer, neural probe array microassembly, microelectromechanical systems

## Abstract

Patients with paralysis, spinal cord injury, or amputated limbs could benefit from using brain–machine interface technology for communication and neurorehabilitation. In this study, a 32-channel three-dimensional (3D) multielectrode probe array was developed for the neural interface system of a brain–machine interface to monitor neural activity. A novel microassembly technique involving lead transfer was used to prevent misalignment in the bonding plane during the orthogonal assembly of the 3D multielectrode probe array. Standard microassembly and biopackaging processes were utilized to implement the proposed lead transfer technique. The maximum profile of the integrated 3D neural device was set to 0.50 mm above the pia mater to reduce trauma to brain cells. Benchtop tests characterized the electrical impedance of the neural device. A characterization test revealed that the impedance of the 3D multielectrode probe array was on average approximately 0.55 MΩ at a frequency of 1 KHz. Moreover, in vitro cytotoxicity tests verified the biocompatibility of the device. Subsequently, 3D multielectrode probe arrays were implanted in rats and exhibited the capability to record local field potentials and spike signals.

## 1. Introduction

Paralysis is a debilitating condition of the neuromuscular system characterized by partial or complete loss of motor functions. There are approximately 5.4 million paralyzed persons in the United States [1]. Traumatic brain injury, spinal cord injury, and stroke are some of the primary conditions that cause paralysis. Brain–machine interfaces can assist patients with paralysis in performing simple tasks, including checking emails, operating a television set [2], and controlling basic robotic arm movements [3,4] by using only their neural activity. This process involves decoding of brain signals and translating these signals into corresponding movements [5]. These signals are then circumvented from the damaged tissue and converted into external control signals.

There are several neural recording devices which use brain–machine interfaces to acquire brain signals. For instance, an electroencephalography (EEG) signal is acquired through the scalp using a non-invasive neural recording device, whereas invasive neural recording devices could acquire an electrocorticography (ECoG) signal from epidural or subdural and local field potentials (LFP), as well as spike signals from motor cortex region [6]. LFP and spike signals of brain activity can be acquired by neural probes, which have different designs and varying numbers of electrodes. Many researchers believed that this type of neural device could capture the most useful human brain signals for more complicated brain–machine interface applications [7]. Prominent extracellular potential is detected upon insertion of neural probes into the motor cortex region. With respect to the measured action potentials (APs), spike signals have a frequency band of 300 Hz to 7 kHz with amplitudes of 10 μV to 500 μV. EEG signals have a frequency band of 1 Hz to 100 Hz with amplitudes of 1 μV to 10 μV [8].

Metal-based [9] and silicon (Si)-based neural probe arrays [10,11] have been extensively developed to monitor neural activity in the brain, particularly in cortical recording and motor prosthesis applications. Floating microelectrode arrays (FMAs) [9] and Utah electrode arrays (UEAs) [10,12] are commercially prominent in neuroscientific research. However, these probes are designed with only one recording electrode at their probe tips because of the limitations of processing technology that these probes apply. Moreover, the spatial resolution of the recording sites of these probes is limited. Only UEAs, which are Si-based, are currently approved for human use despite having a limited number of recording sites. To increase the spatial resolution of recording sites, complementary metal–oxide–semiconductor (CMOS) neural probes have been proposed [13]. These probes have been implemented in 0.18 μm and 0.6 μm CMOS technologies. The use of CMOS integrated circuits (ICs) for probes significantly increased the numbers of recording electrodes to 188 channels and allows an integrated on-shaft circuitry. These probes can provide neural recording performance with a high signal-to-noise ratio, sufficient gain, and a dynamic range. However, this may lead to higher energy consumption as compared to micromachining-based probes. Another drawback for the CMOS–based neural probe may be high overhead cost due to the expensive and sophisticated CMOS IC fabrication process.

There are several three-dimensional (3D) neural probe array assembly and packaging approaches that have already been presented [14,15,16,17,18,19,20]. Barz et al. [14] created 3D probe arrays using stacking modules in between the planar neural probes, while Chang et al. [15] used layer-by-layer spacers between the planar neural probes which can be wire-bonded individually. Wang et al. [16] presented a method where shanks are vertically folded upward from planar probes using heat treatment. Du et al. [17] created 3D electrode arrays using a micromachining process where the recording electrodes are patterned on both surfaces of the planar probe structure. On the other hand, Herwik et al. [18] created 3D arrays by integrating the planar probes with highly flexible ribbon cable to form the electrical interconnection before inserting it into a slim platform. Different ways to form out-of-plane electrical interconnection were also presented. For instance, Aarts et al. [19] used overhanging gold clips on the edge of a cavity of a Si platform to form a mechanical-electrical connection with the perpendicularly assembled probe array. Cheng et al. [20] used Si interposer bonded on the planar probe prior to the insertion into the slot of Si platform. 

In this study, we propose an ultracompact multielectrode three-dimensional (3D) neural probe array that can free float on the brain, thereby preventing contact between the implant and the skull. The maximum profile of the assembled device was kept at less than 0.50 mm above the pia mater to reduce trauma to brain cells. The length and number of electrodes per shank in the probe are customizable; that is, the probe can be designed and fabricated according to individual specifications or requirements. The ultracompact 3D probe array assembly and packaging method are detailed in this paper. Two-dimensional (2D) multielectrode probes are designed to suit a microassembly with a thin Si platform that can be assembled and forms a 3D neural device. The integrated 3D neural device can be connected to a commercially available neural recording instrument for neural monitoring. A cytotoxicity test and functionality experiment were performed in vitro and in vivo to verify the biocompatibility and functionality, respectively. Neural spike signals in animal brain were recorded using the assembled device.

## 2. Materials and Methods

**Figure 1:**
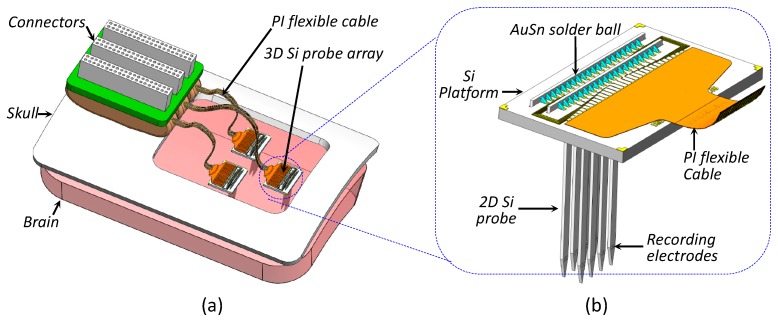
(**a**) Proposed 3D probe array microsystem implanted into the brain (not drawn to scale) and (**b**) magnified view of 3D probe array.

Local field potentials are electrical signals due to the summed activity of multiple neurons in a small volume of nervous tissue that are recorded using implantable probe arrays. LFPs recorded from the ventral hippocampus of urethane-anesthetized rats show characteristic theta oscillations. Action potentials (APs) are also known as nerve impulses or spike signals. The bandwidth and amplitude ranges of APs are 300 Hz to 7 kHz and 10 μV to 500 μV, respectively. The bandwidth range of the LFPs is 1 Hz to 500 Hz, with a typical amplitude range of 10 μV to 5 mV. LFPs are crucial in neuronal communication and computation. APs are primarily used for neural prostheses. Figure 1a displays the proposed 3D 32-channel (32-ch) probe array microsystem, which can be implanted into the brain, particularly the motor cortex region. This ultracompact neural recording device comprises a multielectrode 3D probe array, a polyimide (PI)-based flexible cable, and a printed circuit board with external connectors. The 2D silicon-based probes use multielectrodes to acquire neural signals from the brain. These multielectrodes are orthogonally micro-assembled with the Si platform. A PI flexible cable is placed at one end of the Si platform for neural signals transmission to the external neural recording instrument.

In chronic intracortical implantation, neural probes directly penetrate the brain, and the top part of the neural device over the cortical surface may get stuck to the skull. In such cases, micromotions in the brain damage the adjoining brain tissue. Therefore, the maximum profile of the probe array should be controlled after implantation. Figure 1b exhibits a magnified view of the proposed low-profile probe array for neural implants. The 2D Si probes are partially embedded and fixed in the Si platform slots.

**Figure 2:**
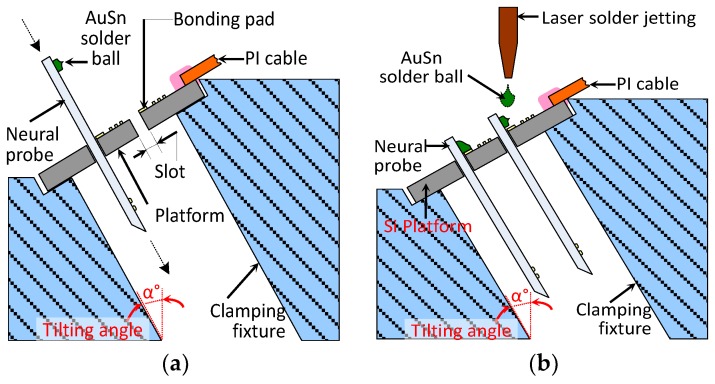
Schematic of proposed lead transfer technique. (**a**) 2D probes orthogonal assembly with Si platform and (**b**) laser solder ball jetting to connect the probes and Si platform.

Lead transfer is a critical interconnection packaging technology used in the 3D microassembly of the probe array. The 2D probes and the Si platform with a flexible PI cable were assembled orthogonally to each other. Lead transfer between these perpendicular conductors was a challenge, as shown in Figure 1b. Figure 2 displays the proposed lead transfer technique. Figure 2a displays the 2D neural probes, which were fabricated using microelectromechanical systems (MEMS) technology. Each probe was designed and fabricated with assembly pins and several bond pads on its backbone.

Gold–tin alloy solder balls (AuSn 80/20 wt%) were attached as solder bumps to the bond pads of the 2D neural probes by using a laser soldering machine. As depicted in Figure 2b, the neural probes with solder bumps were then placed perpendicularly in the Si platform slots. Moreover, the slots maintained the perpendicular alignment between the neural probes’ bond pads and the Si platform. Because the backbone of each probe was partially embedded in the Si platform, the entire probe array profile above the pia mater was minimized. When the probes had been perpendicularly aligned, the AuSn solder balls, which were 120 μm in diameter, were laser-soldered to the orthogonal connection. These solder bumps provided an electrical interconnection between the neural probes and Si platform. A clamping fixture with a tilting angle (α = 30°) was utilized throughout the entire lead transfer process of the solder jetting of the 2D neural probes and Si platform.

**Figure 3:**
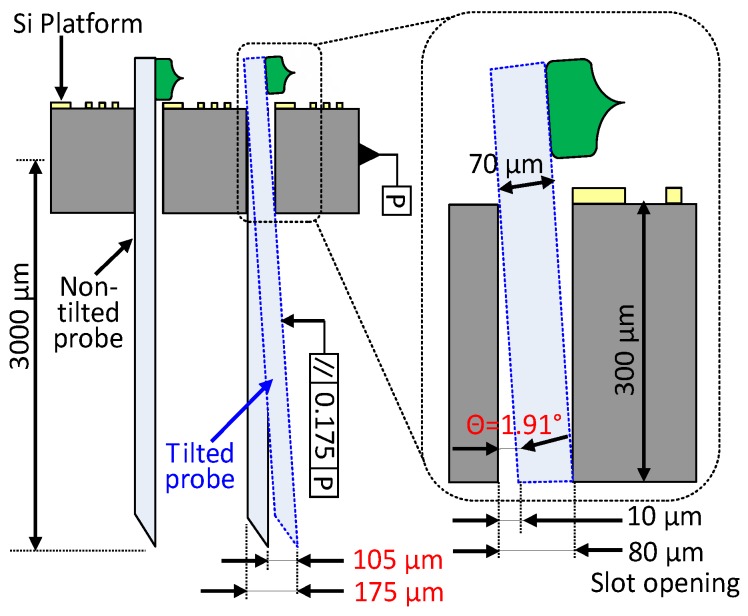
Side view cross-section of assembled probe during assembly misalignment.

Figure 3 exhibits a side view cross-section of the integrated 3D neural probe array and a tilted probe embedded in the Si platform. The thickness of the designed probe was 70 μm. The Si platform had a thickness of 300 μm and a slot opening of 80 μm. The alignment tolerance of the slot opening during probes assembly was 10 μm. Based on these measurements, the approximate maximum angle *θ* of the tilted probe was calculated as follows:

$$\theta =\arctan\left(\frac{80-70}{300}\right)$$

$$\theta =1.91^{\circ }$$

If the length of the assembled probe is 3150 μm and the tilting angle is at its maximum, the tip of the probe shifts by 105 μm, as indicated by the following computation:

$$Probe\;shifting,\;d=\tan1.91^{\circ }\times 3150\;µm$$

d=105 µm

Parallelism of the probe according to the side wall (P) of the Si platform is 175 μm, as shown in Figure 3. Probe misalignment or tilting is likely considerable but can be managed by reducing the tolerance during assembly.

## 3. Fabrication and Packaging

### 3.1. Fabrication

**Figure 4:**
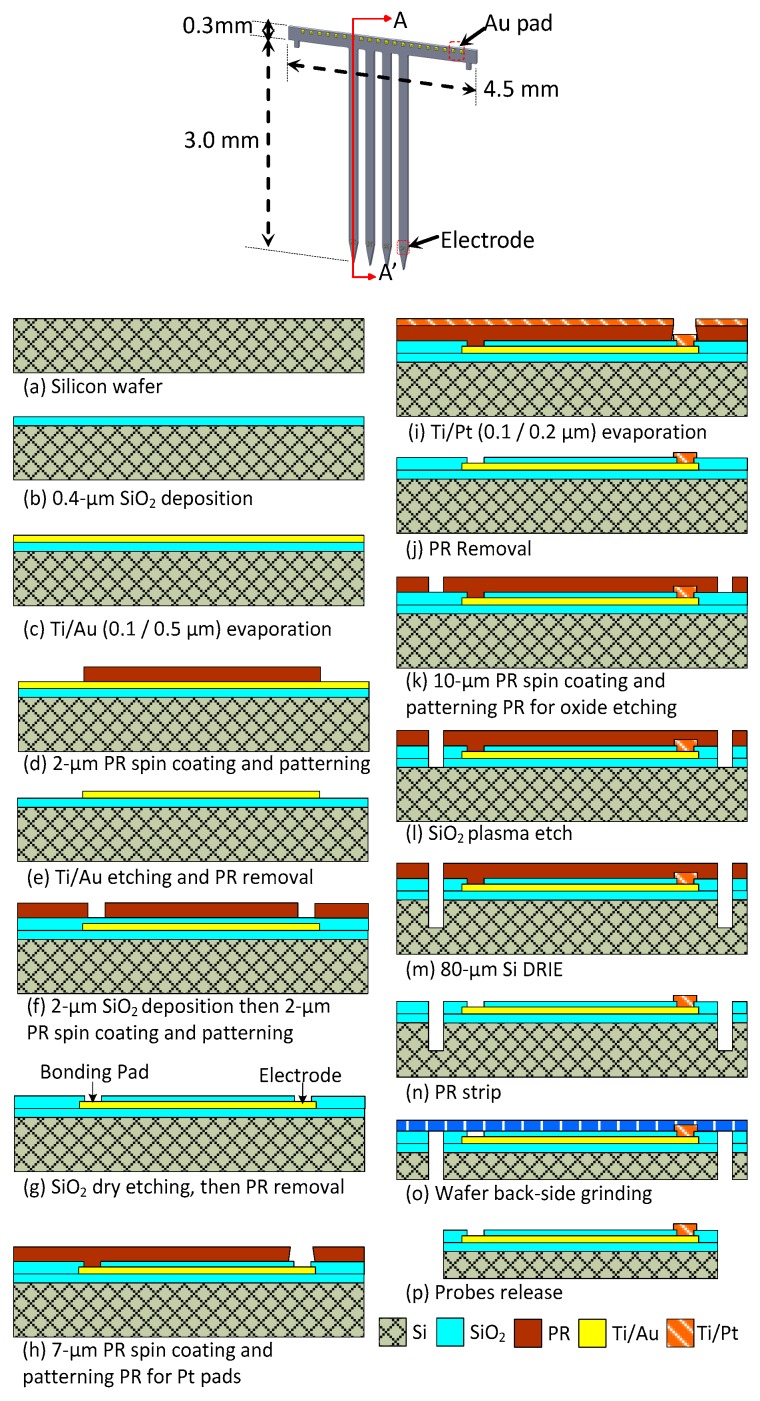
Fabrication flow of 2D probe defined based on section A–A in the 2D probe illustration.

The proposed 3D multielectrode probe array device comprises three parts, namely the 2D probes, Si platform, and flexible PI cable. Figure 4 displays the fabrication flow of the 2D Si probes. Each 2D probe was designed with four shanks and five recording electrodes at each shank. First, a SiO_2_ layer of 4 kÅ was deposited on a silicon wafer as an insulating layer, as shown in Figure 4a,b. This deposition was performed using plasma-enhanced chemical vapor deposition (PECVD). Layers of titanium (Ti) and gold (Au), 1 and 5 kÅ thick, respectively, were then deposited on the wafers through an evaporation process, as shown in Figure 4c. Photoresist (Sumitomo PFI26A) of 2 µm thickness was applied and patterned using a mask on the wafer, as shown in Figure 4d. The electrodes, traces, and bonding pads were defined through the wet etching of Ti and Au, as shown in Figure 4e. A Ti etchant was composed of NH_4_OH, H_2_O_2_, and H_2_O at a ratio of 1:1:3. An Au etchant was composed of HNO_3_, HCl, and H_2_O at a ratio of 1:3:4. Another layer of 2 µm of SiO_2_ was then deposited through the PECVD process, as shown in Figure 4f. To define the bond pads and multielectrodes on the probe, photoresist (Sumitomo PFI26A) of 2 µm thickness was reapplied and patterned on the wafer. A SiO_2_ layer of 2 μm thickness was then deposited on the wafers, as shown in Figure 4f.

Figure 4g illustrates that for bonding pad openings, SiO_2_ was etched using a reactive ion etching (RIE) process that utilizes the Advanced Vacuum RIE Vision 300 MK II machine, as shown in Figure 4g. The optimized process parameters for SiO_2_ etching were as follows: platen power of 300 W and pressure of 100 mTorr. The gas flows of fluoroform (CHF_3_) and oxygen (O_2_) were 45 and 5 sccm, respectively. The SiO_2_ was dry etched at a rate of 330 Ǻ/min. The photoresist was removed after the SiO_2_ was etched using PRS3000 solution from Avantor Performance Materials.

The recording sites of the probes were patterned with platinum (Pt), a low-impedance material, as displayed in Figure 4h–j. Figure 4i exhibits the evaporation of 0.1 μm Ti and 0.2 μm Pt. To define the outline of the neural probe, a 10 µm photoresist (PR) layer was applied and patterned as an etch mask to etch the SiO_2_, as shown in Figure 4k. The RIE process was used to remove the SiO_2_, as displayed in Figure 4l. A deep RIE (DRIE) process with a coil power of 600 W, sulfur hexafluoride (SF_6_) flow rate of 130 sccm, oxygen (O_2_) flow rate of 13 sccm, and octafluorocyclobutane (C_4_F_8_) flow rate of 110 sccm was used to etch Si at a depth of 80 µm. Etching was performed using surface technology systems ICP multiplex etcher, as shown in Figure 4m. The dry etching rate was approximately 2.08 μm/min. The DRIE process and removal of photoresist, shown in Figure 4n, were followed by the backgrinding process illustrated in Figure 4o. Since the probe outline was already defined, it was necessary to use a strong adhesive film and supporting wafer during backgrinding. Ultraviolet (UV) tape was used as an adhesive film to cover the patterned side of the wafer and protect the recording electrodes and bonding pads. The wafers were ground back using a DISCO DGP8761HC wafer grinding machine. The UV-taped wafer was subjected to UV irradiation after the backgrinding process. This procedure ensured that the UV tape was efficiently removed from the patterned side of the wafer. The 2D probes, which were 70 µm thick at that point, were easily released and collected, as shown in Figure 4p.

**Figure 5:**
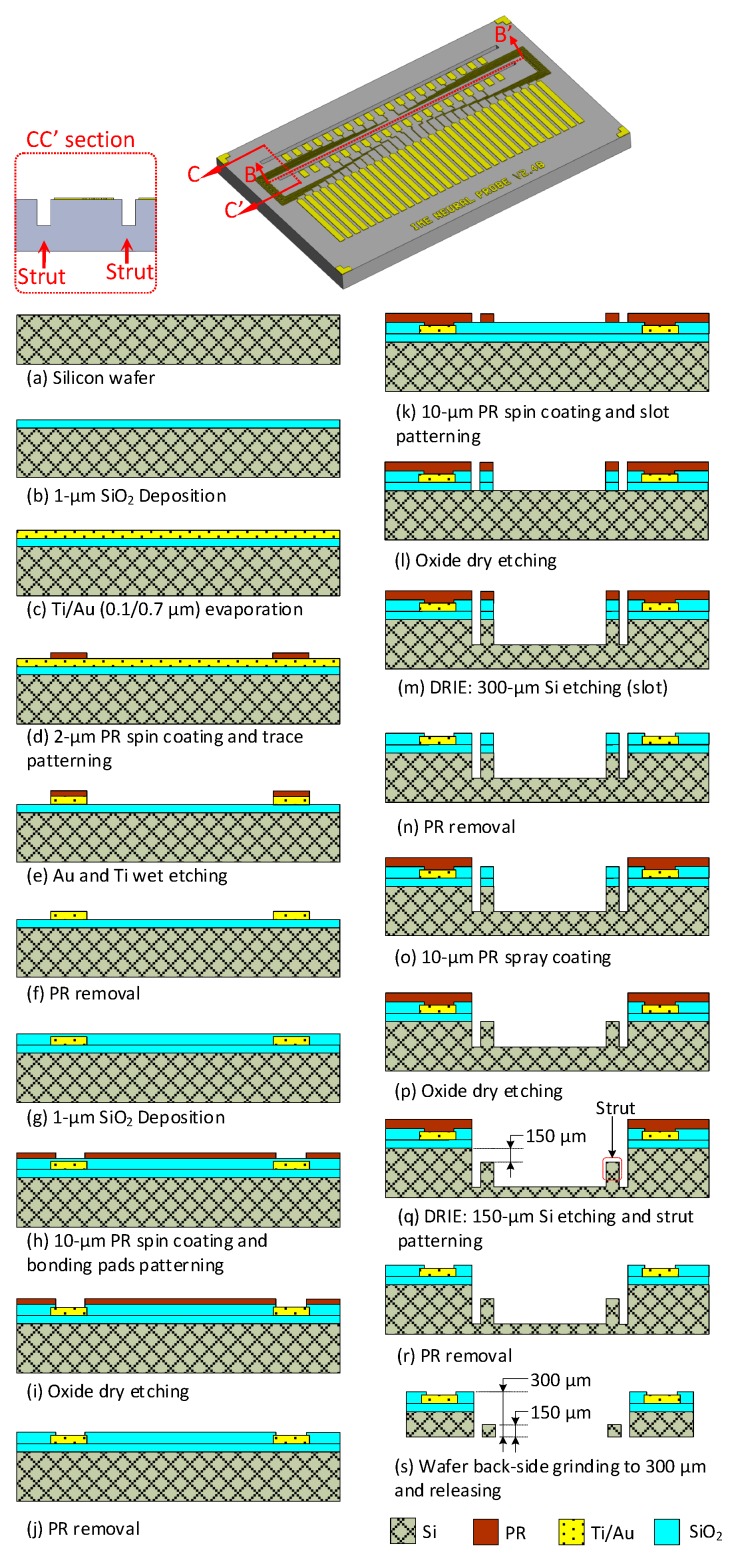
Fabrication flow of silicon platform defined based on section B–B of the illustration.

The Si platform of the 3D multielectrode probe array device served as a carrier for the 2D probes. The platform was designed with two slots of struts for probe assembly and a redistribution layer for interconnection with the flexible PI cable. The fabrication flow of the Si platform fabrication process is shown in Figure 5. First, a 1 µm SiO_2_ layer was deposited on a Si wafer as an insulating layer, as shown in Figure 5a,b. Thin films of 1 kÅ Ti and 7 kÅ Au were deposited on the wafer through a metal evaporation process, as shown in Figure 5c. The wafer was then spin coated with 2 µm PR, as shown in Figure 5d, and the bond pads and traces of the Si platform were patterned using the wet etching process, as shown in Figure 5e. After patterning, PR was removed using the PRS3000 solution, as shown in Figure 5f. A 1 µm SiO_2_ layer was deposited and 10 µm PR was coated using spin coating. The bonding pads and traces of the Si platform were defined and established after oxide etching. At this stage, PR was stripped, as shown in Figure 5g–j.

The slot depth and geometry of the 2D probe integration were defined and created using the DRIE process. A PR layer of 10 µm thickness was coated on the wafer by using SPR220—a coating equipment (Rohm and Haas Electronic Materials)—as shown in Figure 5k. The PR layer served as a mask for etching in the first step of the DRIE process. In the first DRIE procedure, which is illustrated in Figure 5l,m, Si was etched at a depth of 300 µm. The PR was then stripped using the PRS3000 solution, as shown in Figure 5n. To ensure that the 10 μm Clariant AZ5214 negative photoresist coating was uniform, an EVG101 spin or spray coating and developing equipment (EVGroup) were used, as shown in Figure 5o. The oxide layer of the struts was etched through the RIE process, as illustrated in Figure 5p. In Figure 5q, this was followed by a second DRIE process which etched out another 150 µm from the predefined slots of the Si platform. The backbones of the 2D probes were partially embedded at an etching depth of 150 µm, and the fixing slots for the 2D probe assembly pins were established. After this process, the PR was finally removed, as displayed in Figure 5r. The final thickness of the wafer after backgrinding was 300 µm, and thus the slots of the Si platform were exposed. Development of the Si platform was completed after the UV tape had been removed, as shown in Figure 5s.

### 3.2. Packaging

**Figure 6:**
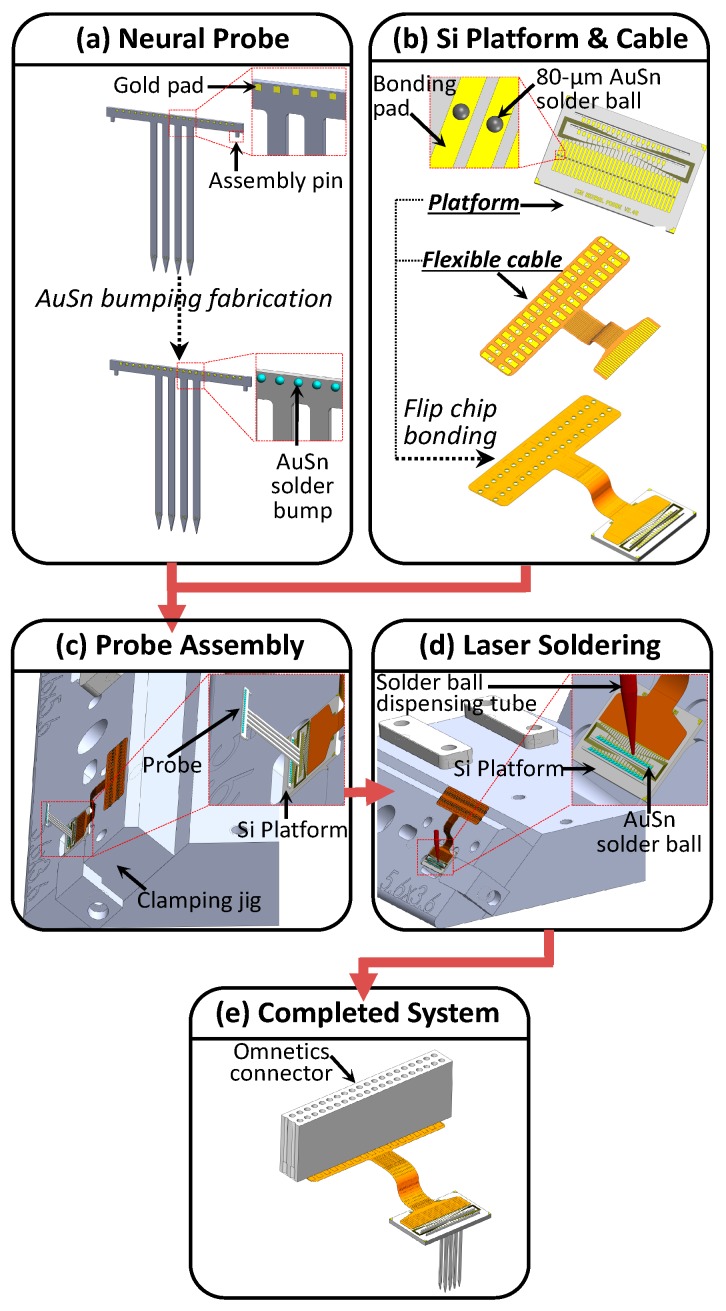
Assembly flow of the 3D multielectrode probe array microsystem.

Figure 6 illustrates the 3D multielectrode probe array device assembly and packaging process. The 2D probe was designed with assembly pins at each end of the backbone. These pins fixed the 2D probes in the struts of the Si platform, thereby creating a 3D structure when assembled together. Before assembling the probe with the Si platform, the bond pads of the 2D probes were attached with AuSn solder balls through a laser solder jetting process, which used the SB2-Jet machine (PAC TECH Packaging Technologies, Inc., Nauen, Germany), as shown in Figure 6a. The optimized solder jetting process parameters were as follows: laser energy of 24 mJ, current of 3500 mA, N_2_ pressure of 40 mbar, and pulse width of 2 ms. Figure 6b shows the integration of the Si platform and flexible PI cable. The Si platform was designed and fabricated with a bond pad space and pitch of 80 and 500 µm, respectively. The bond pads were bumped with AuSn solder balls of 80 µm diameter through the laser solder jetting process. Alongside the aforementioned bond pad space and pitch, the flexible PI cable was bonded to the Si platform by using flip-chip bonder FC150 (Smart Equipment Technology, Saint-Jeoire, France). The optimized flip-chip parameters were as follows: compression force of 800 g and bond temperature of 300 °C for 2 min. Nagase ChemteX UFR115VL underfill—a solvent-free liquid—was used to encapsulate the Si platform prior to flip-chip bonding to prevent electrical shorting between bonding pads.

Figure 6c illustrates the setup of the probe assembly before laser soldering. To fix the Si platform during probe assembly and solder ball jetting, a clamping jig customized according to the size, thickness, and slot location of the Si platform was used. After the Si platform had been fixed, the 2D probes were inserted and aligned in the intended slots. To minimize the probe array profile, the backbones of the 2D probes were only partially embedded in the Si platform, which generated a 3D probe array, as shown in Figure 6c. After embedding, the orthogonally aligned bond pads of both probes and the platform were connected through laser solder jetting, which used AuSn solder balls of 120 µm diameter. This process established the electrical interconnection between the Si platform and 2D probes, as shown in Figure 6d.

To maintain bonding integrity and protect the entire microsystem, a biocompatible UV-cured epoxy Loctite 4305 was coated on the Si platform, flexible PI cable, and 2D probes and then further cured under UV light for 1 min. To complete the 3D probe array microsystem, an Omnetics connector was soldered at the opposite end of the flexible PI cable. The completely assembled 3D neural probe array is shown in Figure 6e. The profile of the 3D multielectrode probe array device is ultracompact at a maximum height of 500 µm above the pia mater.

## 4. Testing Results and Discussion

**Figure 7:**
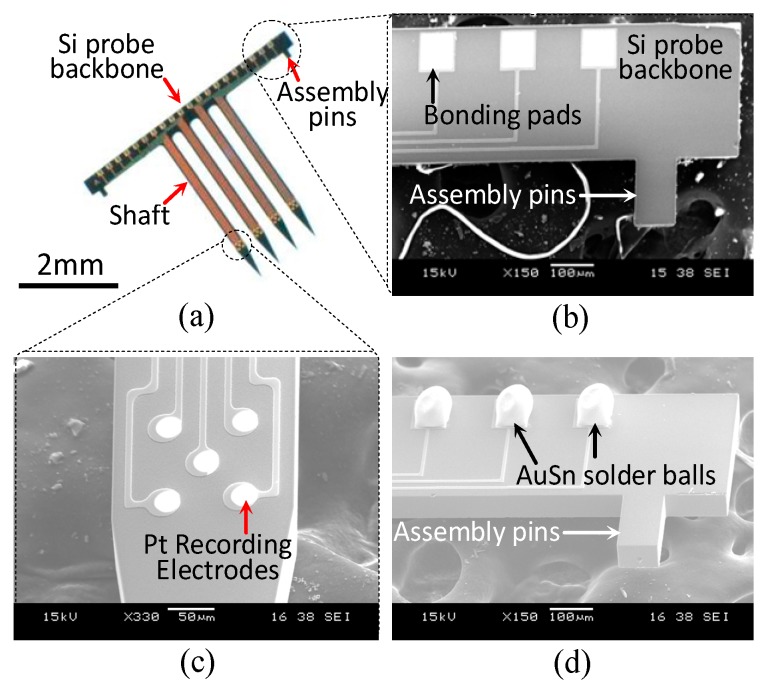
Actual 2D neural probes. (**a**) Probe with 3 mm length shafts and assembly pins at both ends, (**b**) magnified views of probe backbone showing bond pads, (**c**) multielectrode tip, and (**d**) magnified views of probe backbone with AuSn solder ball bumps.

Figure 7 shows the details of the completed 2D neural probe. Figure 7a shows the actual 2D Si probe with four shafts and two assembly pins near the opposite ends of the backbone. The length and width of each shaft are 3 mm and 200 μm, respectively. The details of the backbone of the Si probe are illustrated in Figure 7b. Twenty bonding pads, corresponding to the bond pads on the Si platform for interconnection packaging, were placed on the backbone. The assembly pins anchored the probe to the Si platform. Pt multielectrodes were defined on the tip of each probe shaft, as shown in Figure 7c. To facilitate the packaging and orthogonal assembly with the Si platform, 80 μm AuSn solder balls were attached to the bonding pads of the probe backbone through laser soldering, as shown in Figure 7d.

**Figure 8:**
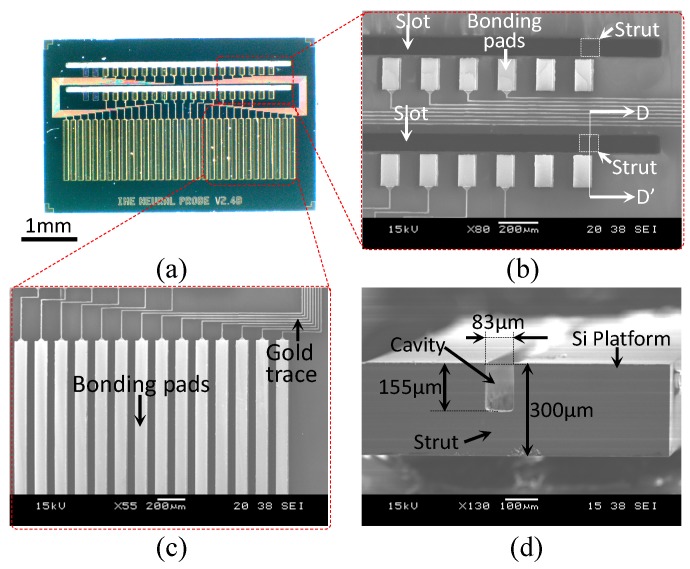
(**a**) Magnified photograph of the fabricated Si platform, (**b**) scanning electron microscopy image of the slots and struts of the Si platform, (**c**) bonding pads of the Si platform intended for flexible PI cable integration, and (**d**) scanning electron microscopy image of section D-D’, showing the cavity and strut of the Si platform (from Figure 8b).

The fabricated Si platform is shown in Figure 8a. The struts of the Si platform were used to support the 2D Si probes when they were slotted into the Si platform to maintain perpendicularity with the platform during assembly, as shown in Figure 8b. Small bonding pads were used for packaging with the Si probes. In Figure 8c, the large-sized rectangular bonding pads of the Si platform were defined for integration with the flexible PI cable. Figure 8d shows a cross-section of the Si platform, which is displayed in Figure 8b (section D-D’) with the detailed dimensions of the strut and cavity.

**Figure 9:**
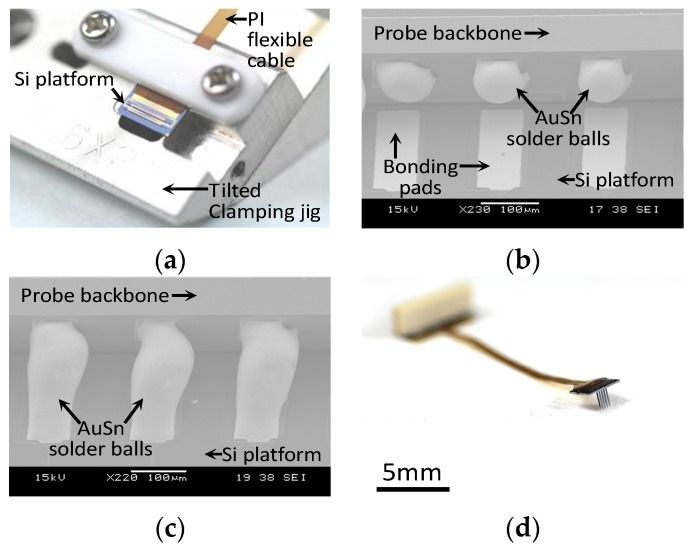
3D probe orthogonal assembly. (**a**) Tilted clamping jig to fix the Si platform during assembly, (**b**) 2D probe slotted into the Si platform before laser soldering, (**c**) laser-soldered junction of the 2D probe and Si platform, and (**d**) assembled 3D probe array neural device.

Figure 9a shows that the device is fixed in the tilted clamping jig for efficient connection between the Si platform and 2D probes during laser soldering. Scanning electron microscopy images before and after laser soldering are shown in Figure 9b,c, respectively. The average diameter and height of the AuSn solder bumps on the probe backbone were 51 and 65 μm, respectively. After the laser soldering process, the AuSn soldering bumps were appropriately connected to the probe and Si platform, as shown in Figure 9c. Figure 9d shows the assembled 32-ch multielectrode probe array.

### 4.1. Benchtop Signal Acquisition and Impedance Measurement of the Assembled Neural Device

**Figure 10:**
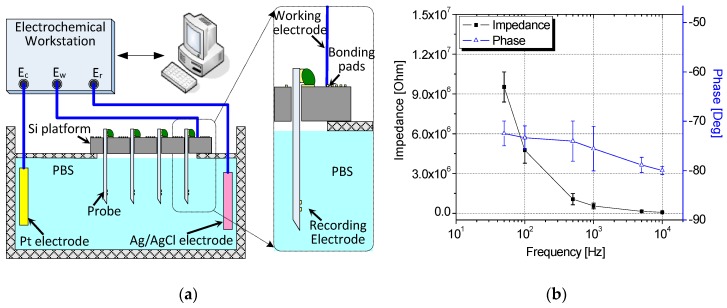
(**a**) Benchtop setup for signal acquisition and impedance measurement and (**b**) measured electrode impedance of assembled probe array.

Figure 10a shows the benchtop setup for signal acquisition and impedance measurement. A custom jig was generated to connect the assembled 3D probe array with a counter electrode (Pt-based electrode) and reference electrode (Ag/AgCl electrode). All electrodes, excluding the bottom surface of the Si platform of the 3D probe array, were submerged in a phosphate-buffered saline solution. The opposite ends of the electrodes were connected to a workstation with a frequency response analyzer. Moreover, a digital multimeter was connected to the setup. In practice, electrode impedance is generally measured at a mean frequency of 1 KHz for neurophysiological activity. This is adequate to specify electrode tip properties [21]. In this set-up, impedance measurement was conducted at a frequency range of 10 Hz–100 KHz. The average impedance measured at 1 KHz was approximately 0.55 MΩ, as shown in Figure 10b.

### 4.2. In Vitro Cytotoxicity Testing Preparation

Before in vivo tests were performed, in vitro cytotoxicity assessment was conducted, primarily to ensure that the assembled neural device would not damage living cells when the probe array is implanted. The methods of cytotoxicity testing and sample preparation were based on the two standard protocols of ISO 10993-5 [22] and ISO 10993-12 [23]. Cell culture NCTC Clone 929 mouse connective tissue cell (ATCC No. CCL-1) was used to evaluate the cytotoxic effect. The cell cultures were incubated at 37 °C for 24 to 48 h until a subconfluent monolayer was formed. For the sample extracts preparation, the neural device samples were prepared in 35 mL of extraction medium, with an extraction ratio of 0.2 g per mL. One piece of 60 cm^2^ high-density polyethylene (HDPE) panel (USP) was used as negative control and 0.016 g Zinc Sulphate (Panreac Quimica Sau, Barcelona, Spain) was used as positive control. The extraction medium used for all samples was Complete MEM10 and the extraction condition was 37 °C for 24 h in a CO_2_ incubator. Sample extracts were filtered by passing through a 0.2 µm membrane filter prior to use for the test. After incubation of the cell culture, portions from the sample extracts replaced the culture medium from the subconfluent monolayer. These were then incubated at 37 °C for 48 h.

### 4.3. Biocompatibility Test

**Figure 11:**
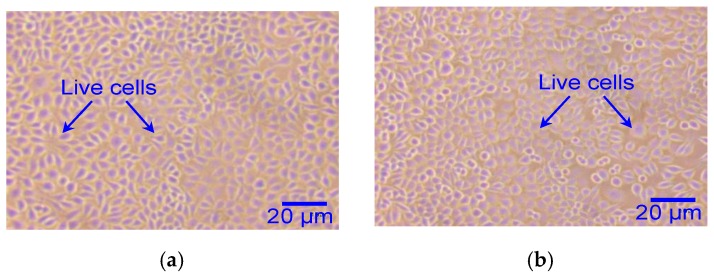

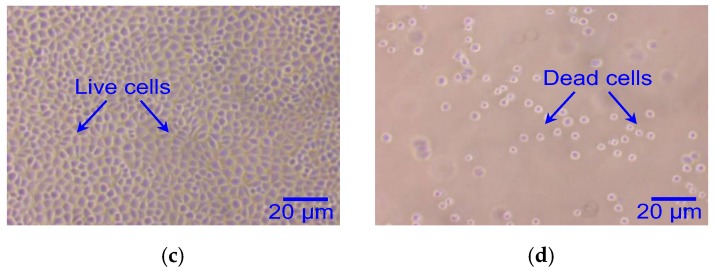
Cell photographs from cytotoxicity assessment. (**a**) S1/sample: 3D multielectrode neural probe array, (**b**) S2/negative control: HDPE, (**c**) S3/reagent blank, and (**d**) S4/positive control: zinc sulfate solution.

All tests were performed in triplicates. After incubation of the cell culture with the sample extracts, the samples were examined microscopically at 100 times magnification. Based on the results, the degree of cytotoxicity of the extracts from the neural device sample, HDPE, and reagent blank was Grade 0; while the positive control is Grade 4. Grade 0 means no reactivity with no destruction of cell layers and Grade 4 means severe reactivity with nearly complete destruction of cell layers. The cells that were extracted from the device shown in Figure 11a were comparable to the negative control, which was extracted from a HDPE material and reagent blank, shown in Figure 11b,c, respectively. The results indicated no cell lysis or no reduction of cell growth, a positive assessment, thereby verifying that the device is biocompatible and has no cytotoxic effect. Figure 11d shows damaged cells from the positive control extract obtained using a zinc sulfate solution.

### 4.4. In Vivo Testing Preparation: Surgery and Electrophysiological Recordings

**Figure 12:**
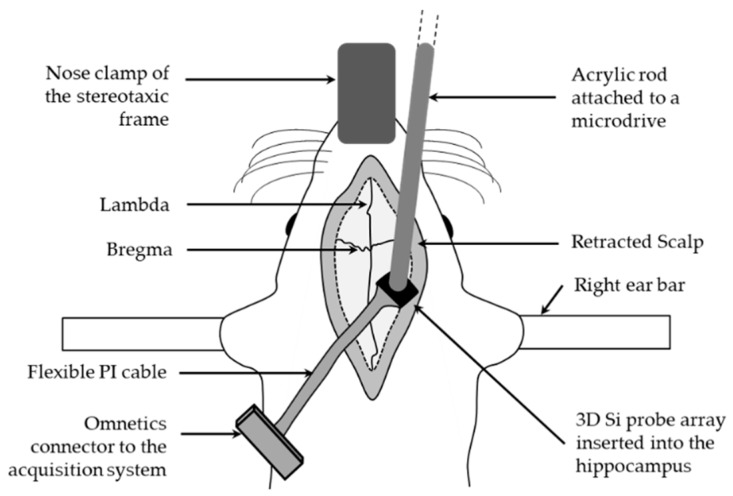
In vivo test setup on urethane-anaesthetized Sprague–Dawley rat mounted on a stereotaxic frame. 3D multielectrode neural probe array inserted into the ventral hippocampus through a burr hole to record the local field potential.

The acute terminal experiments were carried out in 3 Sprague–Dawley rats. Typically, a rat (300–400 g) was anaesthetized with urethane (0.8 g/kg) and mounted on a stereotaxic frame. All animal experiments were approved by the Institutional Animal Care and Use Committee (IACUC), National University of Singapore. A midline sagittal incision was performed, and the scalp was retracted to expose the bare skull. Burr holes were drilled relative to the bregma to target the medial septum (AP:0.2, ML: 0, DV: 5–6 mm) or the ventral hippocampus (AP: −6.5, ML: 5.5, DV: 5.5–6 mm). The 5-shank electrode assembly that was attached to an acrylic rod which in turn was held by a microdrive. The electrode was carefully inserted into the burr hole and then gradually advanced in micrometer steps into the brain to record either the LFP or spike activity similar to previously published procedures [24]. The experimental set up is shown in Figure 12. LFPs from the ventral hippocampus were simultaneously recorded from 2 channels using a preamplifier (EX4-400, Dagan Corporation, USA), digitized (CED, UK), and visualized using Spike 2 (version 7.12). The raw waveforms recorded from the ventral hippocampus were subjected to standard DC noise removal and smoothing algorithms provided by the Spike 2 software. Spike activity from medial septum was acquired using a preamplifier (ELC-03XS; NPI electronics; Tamm, Germany), digitized (Power 1401; CED, Cambridge, UK), and visualized using Spike 2 (version 7.12). Spikes were sorted according to template shape, principal component, density plot, and interspike interval histogram analysis.

### 4.5. In Vivo Test

**Figure 13:**
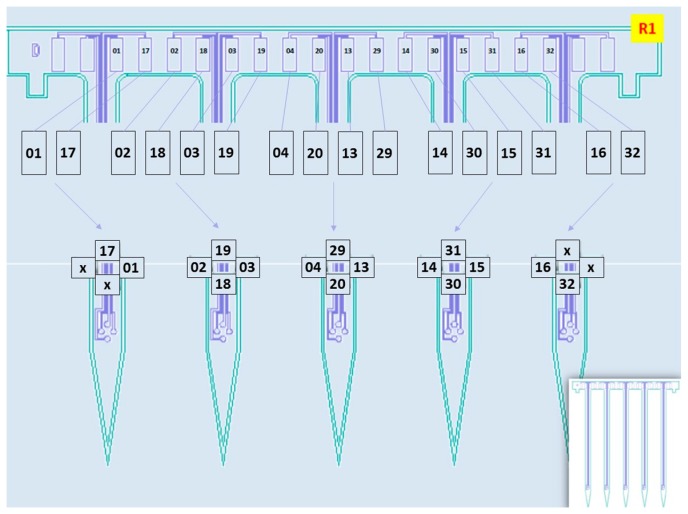
Electrode sites configuration of the planar probe with four recording sites per shank.

**Figure 14:**
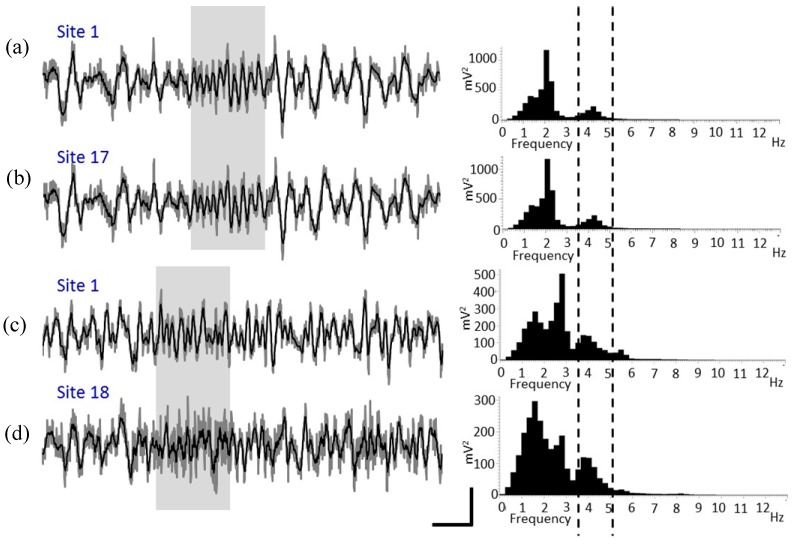
Representative plots of theta oscillation from ventral hippocampus of a urethane-anesthetized Sprague–Dawley rat. The left panel (**a**–**d**) shows raw (dark gray line) and smoothed (black line) waveforms from corresponding recording sites (Scale bar: 200 mV and 1 s), and the right panel shows FFT power spectrums generated for 150 s of data. The light-gray box in the left panel and the broken lines in right panel depict the characteristic theta oscillations caused by urethane.

**Figure 15:**
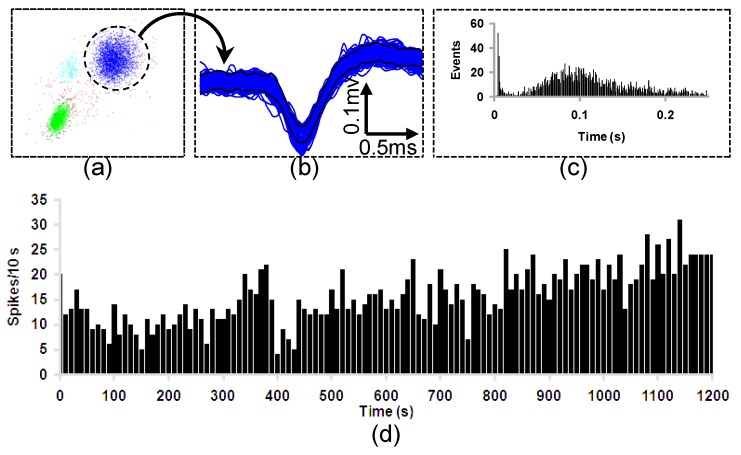
Representative plot of spike recordings from the medial septum of an urethane anesthetized Sprague-Dawley rat. (**a**) Principal components analysis of the entire neuronal events. (**b**) The spike overlay (Scale bar: 0.1 mV and 0.5 ms) of the reliable principal component (in the dotted circle) representing a burst firing neuron of the medial septum with the (**c**) characteristic interspike interval histogram. (**d**) Firing rate (per 10 s) of the neuron for a duration of 1200 s.

To further validate the functionality of the 3D multielectrode probe array, an in vivo test was performed on Sprague–Dawley rats (Figure 12). Electrode sites configuration of the planar probe was shown in Figure 13. The characteristic urethane-induced theta oscillations of the LFP in the ventral hippocampus were simultaneously recorded from multiple sites of the electrode array (Figure 14). The synchrony in the recorded theta oscillations from sites 1 and 17 (Figure 14a,b) indicate that the field potentials were recorded from sites close to each other in the same shank (Figure 13). Likewise, the theta oscillations from sites 1 and 18 (Figure 14c,d) were not synchronous, indicating that the sites of recording are far from each other, different shanks in this case (see Figure 13). Furthermore, stable recording of negative-going spikes from the medial septal region were obtained for a period of 20 min (Figure 15d). Following the data acquisition, the spikes sorted using the principal component analysis (Figure 15a,b) were observed to show the burst firing type from the interspike interval histogram (Figure 15c).

## 5. Conclusions

The orthogonal microassembly and ultracompact packaging of the proposed 32-ch 3D multielectrode neural probe array device are presented in this paper. The thickness of each 2D multielectrode probe was reduced to 70 μm to fit the slots of the Si platform. The Si platform has struts that facilitated a minimum profile of the probe array after assembly and are designed with bond pads for electrical interconnection between the 2D probes and ultrathin flexible PI cable. Proposed micromachining and packaging processes enable the orthogonal microassembly of the 2D probes and Si platform. The maximum profile of the probe array of the assembled device is 500 μm above the pia mater. The benchtop test of the assembled probe array in acquiring signals verified the functionality. The measured impedance, on average, was approximately 0.55 MΩ at a frequency of 1 kHz. The in vitro cytotoxicity assessment verified that the proposed integrated neural probe array microsystem is biocompatible. The in vivo test further verified the functionality of the neural device in recording LFPs and spike activity from a urethane-anesthetized Sprague–Dawley rat. The characteristic urethane-induced theta oscillations of the LFP in the ventral hippocampus were simultaneously recorded from multiple sites of the electrode array.
